# Ethanolic Extract of *Centella asiatica* Treatment in the Early Stage of Hyperglycemia Condition Inhibits Glomerular Injury and Vascular Remodeling in Diabetic Rat Model

**DOI:** 10.1155/2021/6671130

**Published:** 2021-07-06

**Authors:** Wiwit A. W. Setyaningsih, Nur Arfian, Akbar S. Fitriawan, Ratih Yuniartha, Dwi C. R. Sari

**Affiliations:** ^1^Department of Anatomy, Faculty of Medicine, Public Health, and Nursing, Universitas Gadjah Mada, Yogyakarta 55281, Indonesia; ^2^Department of Nursing, Faculty of Health Sciences, Universitas Respati Yogyakarta, Yogyakarta 55282, Indonesia

## Abstract

**Background:**

Diabetes mellitus (DM) is marked by oxidative stress, inflammation, and vascular dysfunction that caused diabetic nephropathy that resulted in end-stage renal disease (ESRD). Vascular dysfunction is characterized by an imbalance in vasoconstrictor and vasodilator agents which underlies the mechanism of vascular injury in DM. Additionally, diminished podocytes correlate with the severity of kidney injury. Podocyturia often precedes proteinuria in several kidney diseases, including diabetic kidney disease. *Centella asiatica* (CeA) is known as an anti-inflammatory and antioxidant and has neuroprotective effects. This research aimed to investigate the potential effect of CeA to inhibit glomerular injury and vascular remodeling in DM.

**Methods:**

The DM rat model was induced through intraperitoneal injection of streptozotocin 60 mg/kg body weight (BW), and then rats were divided into 1-month DM (DM1, *n* = 5), 2-month DM (DM2, *n* = 5), early DM concurrent with CeA treatment for 2 months (DMC2, *n* = 5), and 1-month DM treated with CeA for 1-month (DM1C1, *n* = 5). The CeA (400 mg/kg BW) was given daily via oral gavage. The control group (Control, *n* = 5) was maintained for 2 months. Finally, rats were euthanized and kidneys were harvested to assess vascular remodeling using Sirius Red staining and the mRNA expression of superoxide dismutase, podocytes marker, ACE2, eNOS, and ppET-1 using RT-PCR.

**Results:**

The DM groups demonstrated significant elevation of glucose level, glomerulosclerosis, and proteinuria. A significant reduction of SOD1 and SOD3 promotes the downregulation of nephrin and upregulation of TRPC6 mRNA expressions in rat glomerular kidney. Besides, this condition enhanced ppET-1 and inhibited eNOS and ACE2 mRNA expressions that lead to the development of vascular remodeling marked by an increase of wall thickness, and lumen wall area ratio (LWAR). Treatment of CeA, especially the DMC2 group, attenuated glomerular injury and showed the reversal of induced conditions.

**Conclusions:**

*Centella asiatica* treatment at the early stage of diabetes mellitus ameliorates glomerulosclerosis and vascular injury via increasing antioxidant enzymes.

## 1. Introduction

Diabetic nephropathy (DN) is one of the late complications of the diabetes mellitus (DM) and results in end-stage renal disease (ESRD) [1, 2]. It is widely known that uncontrolled chronic hyperglycemia disturbs the mitochondrial electron-transport chain producing superoxides. An excess of superoxides enhances the alteration of various pathways such as protein kinase-C (PKC) pathways, advanced glycation end (AGE) product, hexosamine, and polyol pathways [3]. The formation of superoxides leads to an increase of reactive oxygen species (ROS) which causes oxidative stress, inflammation, and vascular complications [4, 5]. It is widely reported that the plasma level of superoxide dismutase (SOD) and glutathione peroxidase (GSH-Px) were lower in the diabetic condition [6, 7] which increases the risk of cardiovascular disease. Superoxide dismutase catalyzes superoxide anion (O_2_^−^) into hydrogen peroxide; however, the high glucose level inside the cells promotes production of ROS. There are three types of SOD: (1) SOD1 (Cu/Zn SOD) or intracellular SOD found in the cytoplasm and nuclei, (2) SOD2 (MnSOD) localized in the mitochondrial matrix, and (3) SOD3 (ZnSOD) or extracellular SOD found in the vascular extracellular space and highly expressed in the blood vessels and heart [8, 9].

Podocyte loss is the early injury in DN, then resulting in decreasing podocyte density and increasing albuminuria [10, 11]. Podocyte injury occurs since the early stage of DM and it dramatically worsens with the progressivity of diabetes. Podocyte loss also induces downregulation of podocyte protein, such as WT-1, that undergoes shifting localization of from the nucleus to cytoplasmic [12], and nephrin, an adhesion protein between the foot processes of the podocyte. At the early stage of glomerular injury, the urinary expression of nephrin can be detected before the proteinuria [13, 14]. Furthermore, in the glomeruli of patients with diabetes, the protein expression of nephrin reduces compared to the control subjects [15, 16]. Hyperglycemic itself may induce activation of the transient receptor potential canonical channel C6 (TRPC6), a receptor-operated cation channel, which is expressed in podocytes, mesangial cells, and endothelial cells; several conditions produce different results in TRPC6 expression. Downregulation of TRPC6 occurs in mesangial cells culture under high glucose treatment [17, 18]. On the other hand, several studies suggested that the treatment of high glucose increases TRPC6 in both the glomerulus and the heart which associated with ROS production and the renin-angiotensin system (RAS) activation [14, 19].

DM is often associated with microvascular and macrovascular diseases, as complication of the early to late hyperglycemia condition [20, 21]. Chronic hyperglycemia activates RAS that contributes to the glomerular hypertension exacerbating endothelial dysfunction. However, this mechanism is not fully understood [22]. Endothelin-1 (ET-1) has been known as a potent vasoconstrictor which promotes the imbalance between vasoconstrictor and vasodilator substances [23]. Diminished production of nitrite oxide (NO) contributes to the progressivity of cardiovascular and kidney damage mediated by impairment of vascular damage [24–26]. Plasma ET-1 and microangiopathy are positively correlated with the severity of type 2 DM that leads to vascular dysfunction [27]. Imbalance in vasoactive substances and local growth factors contribute to the pathophysiology of vascular injury [28]. Moreover, activation of vasoactive, such as RAS, induces oxidative stress which promotes vascular injury and remodeling that alter lumen and wall areas [28]. The essential landmark for the new concept of RAS is the characterization of angiotensin 1–7 (Ang-1-7) produced by ACE2 which has a vasodilatation effect [29]. On the other hand, ET-1 also contribute to vascular remodeling of intrarenal arteries in kidney ischemic/reperfusion injury [30].

*Centella asiatica* (CeA) is an herbaceous plant that is found in tropical climate countries. It has been widely used as a traditional herbal medicine due to its anti-inflammatory, antioxidant, and neuroprotective effects [31–34]. The main components of CeA are triterpenoids that consist of asiatic acid, madecassic acid, asiatocoside, and madecassicoside. Madecassic acid-contained CeA demonstrated antidiabetic effects through diminished ROS, increased catalase and glutathione, and reduced inflammatory processes [34, 35]. In addition, madecassic acid has an important role as the activator of peroxisome proliferator-activated receptor *γ* (PPAR*γ*) through binding in the 5′ upstream element of peroxisome proliferator response element (PPRE). This process enhances the sensitivity of insulin even though this process remains unclear. PPAR*γ* is widely found in the adipose tissue, intestines cells, macrophages, and endothelial cells. Once it is activated, agonist PPAR*γ* promotes inhibition of tumor necrosis factor-alpha (TNF-*α*), nuclear factor kappa-beta (NF*κ*B), and activator protein-1 (AP-1). Unless, madecassic per se induces the elevation of low-density lipoproteins (LDL) through upregulation of CD36 [34, 36, 37].

In this study, we aimed to elucidate role of CeA treatment either at the early or late hyperglycemia condition of DM. We focused on the glomerular injury and vascular remodeling that are mediated by its antioxidant properties.

## 2. Materials and Methods

### 2.1. Animal Experiment and Kidney Harvesting

The DM rat model was performed through intraperitoneally injection of streptozotocin (60 mg/kg body weight (BW)) single dose [38, 39]. Wistar male rats (3 months old, 160–200 grams) were then divided into DM for 1 month (DM1, *n* = 5), DM 2 months (DM2, *n* = 5), DM-treated CeA for 2 months started from the first diagnosis of DM (DMC2, *n* = 5), and DM-treated CeA after one month of DM (DM1C1, *n* = 5). The ethanolic extract of CeA (400 mg/kg BW) was given daily via oral gavage. At the end of study (after 2 months), rats were anesthetized with ketamine (50 mg/kg BW), xylazine (2 mg/kg BW), and acepromazine (0.5 mg/kg BW). Then, the abdomen and thorax were opened to access the heart and kidney. Next, the organs were perfused using NaCl 0.9% from left ventricle. Finally, the kidney was harvested, the left kidney was immersed in RNA Later (Ambion, AM7021), and the right kidney was kept in neutral buffer formalin (NBF).

This research was conducted according to the guidelines for animal care of the Universitas Gadjah Mada and had been approved by the Medical and Health Research Ethics Committee of the Faculty of Medicine, Public Health, and Nursing, Universitas Gadjah Mada, with ethical expediency number KE/FK/1211/EC/2019.

### 2.2. *Centella asiatica* Extraction

The CeA leaves were attained from Merapi Herbal Farma (the commercial herbal manufacturer) and the isolation method has been described according to the previous study [40]. *Centella asiatica* (400 mg/kg BW) [41] was administered via oral gavage for 1 month and 2 months after diabetes mellitus induction. This process was performed in the Department of Pharmacology and Therapeutics, Faculty of Medicine, Public Health, and Nursing, Universitas Gadjah Mada.

### 2.3. RNA Extraction and cDNA Synthesis

Kidney was extracted using Genezol RNA Solution (GENEzol™, GZR100) according to the protocol from the manufacturer. The RNA concentration was quantified using a nanodrop (Maestrogen, MN-913A). The RNA was synthesized into cDNA using cDNA synthesis kit (SMOBio, RP1400) with PCR condition: 30°C for 10 min., 42°C for 60 min., and 85°C for 5 min.

### 2.4. Reverse Transcriptase-PCR (RT-PCR)

Reverse Transcriptase-PCR was performed to amplify several specific target genes which consisted of antioxidant enzymes, nephrin, TRPC6, ACE2, eNOS, and ppET-1 using the following primers' sequences (Table 1):

### 2.5. Vascular Remodeling

The kidney-embedded paraffin was cut into 4 *µ*m in thickness followed with deparaffinized using xylene and rehydrated using 100%, 90%, 80%, and 70% ethanol. Afterwards, slides were incubated using Sirius Red for an hour to assess wall thickness in intrarenal arteries (diameter 10–50 *μ*m) [30]. Finally, the slides were captured using Optilab software in 400x magnification of 15 random areas.

### 2.6. Immunohistochemical (IHC) Staining of SOD-1 and WT-1

The slides were deparaffinized using xylene and rehydrated using 100%, 90%, 80%, and 70% ethanol which was then followed with antigen retrieval using citrate buffer pH 6 and blocking peroxidase using H_2_0_2_ 3% in PBS solution. Afterwards, slides were incubated with blocking serum (Finetest, IHC007), and rabbit 1^st^ monoclonal antibody SOD1 (1 : 100, Bioss, bs-10216R) and WT-1 (1 : 50, Santa Cruz, sc-192) overnight. Finally, the slides were incubated with poly-HRP Goat-anti rabbit and diaminobenzidine tetrahydrochloride (DAB) (Finetest, IHC007). The results were assessed with light microscope (Olympus, CX22^®^), and captured with Optilab software with 400x magnification.

### 2.7. Western Blot

The kidney was extracted according to the protocol from Pro-Prep^TM^ (Intron Biotechnology, 17081). Twenty milligrams of kidney was homogenized with 600 *μ*L of Pro-Prep^TM^ solution. Then, the homogenates were centrifugated in 15,000 rpm at 4 °C for 20 min. Afterwards, the supernatants were separated onto 10% SDS-PAGE and transferred to a polyvinylidene fluoride (PVDF) membrane. Next, it was incubated with the primary antibodies, *β*-actin (1 : 1000, Abcam, ab8227) and SOD1 (1 : 200, Bioss, bs-10216R) overnight and ended with visualization using ECL Prime Western Blotting Detection Reagents (GE Healthcare, RPN2232) under Geldoc machine (Geldoc Syngene Gbox Seri Chemi xrq).

### 2.8. Statistical Analysis

The SPSS 23 software (IBM Corp., Chicago) for Windows was used for analyses data. Data normality test was conducted using Shapiro–Wilk and one-way ANOVA for normal data distribution. The *p*-value less than 0.05 (*p* < 0.05) was considered statistically significant.

## 3. Results

### 3.1. Depletion of Glucose Level, Proteinuria, and Glomerulosclerosis in CeA-Treated Groups

We demonstrated that streptozotocin injection consistently elevated glucose level compared to the control group (*p*≤0.001), and it lowered when the CeA was administered. The CeA treatment at the early stage of DM (DMC2) significantly reduced glucose level compared to the DM2 (*p* = 0.009). Otherwise, the DM1C1 did not show any significant reduction of glucose level compared to DM groups. The proteinuria was obviously seen in the DM2 group compared to the control group (*p*≤0.001) and DM1 group (*p*≤0.001). The CeA treatment groups, either DMC2 or DM1C1, demonstrated a significant reduction of the proteinuria compared to the DM2 (*p*≤0.001).

The histological staining of glomerulosclerosis demonstrated normal morphological features in the control group; however, the DM groups showed sclerosis, synechiae, thickening of the basement membrane, and narrowing of the glomerular arteriolar. When the CeA was administered, these injuries significantly ameliorated compared to the control group.

### 3.2. *Centella asiatica* Elevated Superoxide Dismutase

The mRNA expression of SOD1 and SOD3 significantly lowered in the DM groups; however, there was no alteration of SOD2 mRNA expression. The mRNA expression of SOD-1 was sharply reduced in the DM1 (*p* = 0.005) and DM2 (*p*≤0.001) compared to the control. The DMC2 showed a significant elevation of the mRNA expression of SOD1 compared to the DM2 (*p* = 0.006) while the DM1C1 showed no difference with the DM groups. We also demonstrated that the SOD1 protein expression decreased in the DM groups compared to the control group. The DMC2 exhibited a significant elevation of the SOD1 protein expression. The SOD1 protein expression reduced sharply in the DM1 (*p* = 0.000), DM2 (*p* = 0.006), DMC2 (*p* = 0.004), and DM1C1 (*p* = 0.000) groups compared to the control group. Administration of the CeA at the DMC2 (*p* = 0.015) improved SOD1 protein expression compared to the DM1 group.

Hyperglycemia-caused DM enhanced a significant reduction of the SOD3 in both DM1 (*p* = 0.004) and DM2 (*p* = 0.003) groups compared to the control group. Besides, the DMC2 demonstrated a significant upregulation of the SOD3 mRNA expression compared to the DM1 (*p* = 0.017), DM2 (*p* = 0.010), and DM1C1 (*p* = 0.038).

### 3.3. CeA Treatment May Associate with High Nephrin and Low TRPC6 mRNA Expressions

Next, our findings suggested that the mRNA expression of TRPC6 was higher in DM1 (*p* = 0.002) and DM2 (*p* = 0.001) groups compared to the control group. Meanwhile, only the DMC2 (*p* = 0.005) had lowered the mRNA expression of TRPC6 compared to the control group. The nephrin was significantly lowered in the DM2 group compared to the control (*p*≤0.001) and DM1 groups (*p* = 0.001). The CeA groups elicited higher nephrin mRNA expression compared to the control group (*p* = 0.001). Lower nephrin mRNA expression aligned with reduction WT-1 protein expression in the kidney and CeA treatment restored the WT-1 protein expression in the glomerulus.

### 3.4. CeA Reduced ppET-1 and Increased Both eNOS and ACE2 mRNA Expressions

Then, we assessed the imbalance of vasoconstrictor and vasodilator agents that promotes vascular remodeling induced by DM. Early stage of hyperglycemia (DM1 group) increased eNOS mRNA level (*p* = 0.045) compared to the control group. Then, the eNOS mRNA expression in DM2 group plummeted (*p* = 0.014) compared to the control group. The eNOS mRNA expression increased significantly in DM1C1 (*p* = 0.007), and DMC2 (*p* = 0.017) compared to the DM1 group as well as DMC2 (*p* = 0.038) compared to the DM2 group. The vasoconstrictor agent, ppET-1, was significantly higher in DM1 (*p*≤0.001) and DM2 (*p*≤0.001) groups compared to the control group. CeA treatment at the early stage of hyperglycemia-induced DM significantly reduced ppET-1 mRNA expression compared to the DM1 (*p* = 0.019) and DM2 (*p* = 0.001) groups. Besides, we assessed the mRNA expression of ACE2 as the counter-arm of angiotensin II. The DM stimulated downregulation of the ACE2 mRNA expression that was obviously seen in DM2 (*p*≤0.001) compared to the control group. However, neither DMC2 (*p* = 0.028) nor DM1C1 (*p*=0.009) promoted significantly higher ACE2 compared to the DM2 group.

### 3.5. *Centella asiatica*-Treated Diabetes Mellitus Ameliorated Vascular Remodelling

Finally, we demonstrated that CeA inhibited vascular remodeling in the kidney which delineated an increase of wall thickness and lumen wall area ratio (LWAR) in the diabetes mellitus groups. Thickening of the vascular wall was obviously seen in DM2 (*p*<0.01) compared to the control and DM1 groups. Treatment of CeA markedly reduced the wall thickness in DMC2 compared to the DM2 (*p* = 0.0255) group as well as in DM1C1 compared to the DM1 (*p* = 0.0219) group. The lumen wall area ratio increased significantly in DM1 (*p* = 0.005) and DM2 (*p* = 0.039) groups compared to the control group. Treatment of CeA in the early stage of hyperglycemia-induced diabetes mellitus (DMC2, *p* = 0.040) remarkably reduced the lumen wall area ratio compared to the DM1 group.

## 4. Discussion

**Figure 1:**
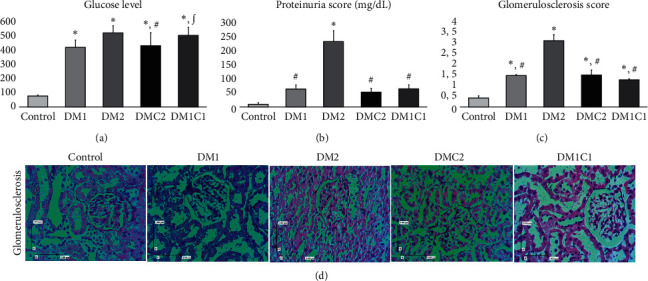
Ethanolic extract of *Centella asiatica* alleviated glucose level, proteinuria, and glomerulosclerosis under DM. (a–c) The results of glucose level, proteinuria, and glomerulosclerosis score. (d) The representative figure of glomerulosclerosis (magnification 400X; scale bar 100 *μ*m). ^*∗*^*p* < 0.01 vs. control, ∫*p* < 0.01 vs. DM1, and ^#^*p* < 0.01 vs. DM2.

This study reveals the protective effect of CeA extract in the early hyperglycemia condition, but not in late hyperglycemia condition in kidney injury as DM progression. CeA treatment in DMC2 group which represented the early CeA treatment, as early as hyperglycemia occurred, may attenuate the diabetic nephropathy (DN). DN initiates chronic kidney disease (CKD) and ESRD that exacerbates the mesangial cell expansion, thickening of the glomerular basement membrane (GBM), and glomerulosclerosis [40, 41]. Since the glomerular filtration barrier (GFB) consists of endothelial cells, GBM, and slit diaphragm (SD), the impairment of the layers contributes to the proteinuria [42]. In this study, diabetes kidney disease was induced through a single intraperitoneal injection (60 mg/kg BW) which resulted in an elevation of blood glucose level and kidney impairment showed by a high level of proteinuria and glomerulosclerosis (Figure 1). High glucose level resulted in damage to the endothelial and mesangial cells due to glucose flooding inside the cells [43]. Lower blood glucose level was associated with CeA treatment in the early-hyperglycemic condition, but not in late hyperglycemic state (Figure 1). This finding demonstrated that attenuation of injury might partially have association with blood glucose reduction.

The CeA treatment significantly reduced the proteinuria and glomerulosclerosis compared to the DM2 group (Figure 1). Oral administration of CeA, both 500 mg/kg BW and 1000 mg/kg BW, showed antihyperglycemic effect that is mediated by *α*-amylase and disaccharidase enzymes inhibition in the intestines [39]. Then, it attenuates blood glucose level and lipid profile serum followed by diminished polyphagia, polydipsia, and polyuria [38, 42]. A previous study mentioned that CeA has several potent active compounds such as asiatic acid, asiatocoside, madecassic acid, madecassoside, astragaloside, and triptolide [33, 44, 45] that cause a reduction in proteinuria and glomerulosclerosis. In addition, the administration of the asiatocoside and asiatic acid significantly reduced urinary protein excretion and blood glucose level in DM rat models [33, 45]. Elucidating active compound in this study might give better understanding for the reno-protective effect of CeA in the future.

**Figure 2:**
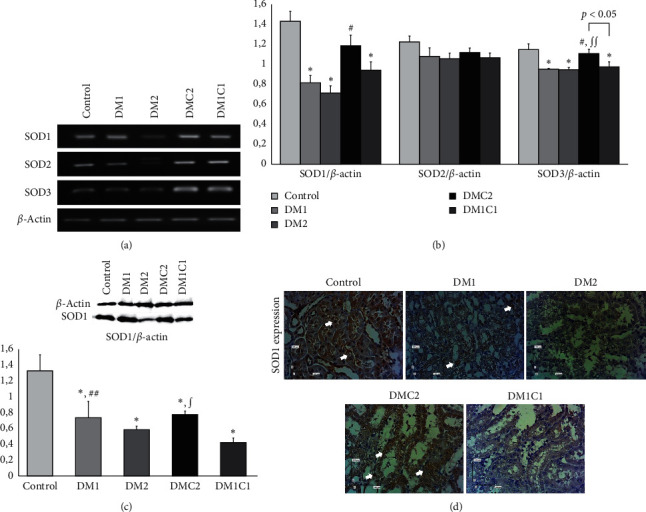
Ethanolic extract of *Centella asiatica* enhanced SOD1 and SOD3 mRNA expressions. (a, b) The representative figures of SOD1, SOD2, and SOD3 mRNA expressions according to the RT-PCR. (c) The representative images of SOD1 protein expression (magnification 400x; scale bar 100 *μ*m). ^*∗*^: <0.01 vs. control, ^∫^: <0.01 vs. DM1 and <0.05 vs. DM1, and ^#^: <0.01 vs. DM2.

**Figure 3:**
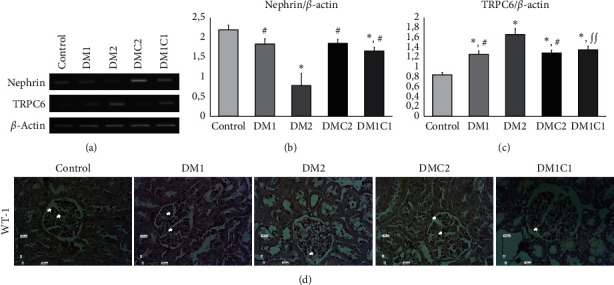
*Centella asiatica* upregulated nephrin and downregulated TRPC6 mRNA expressions with preservation of WT-1 protein staining. (a) The representative pictures of nephrin and TRPC6 mRNA expression based on RT-PCR. (b, c) The bar charts of semiquantitative analysis of nephrin and TRPC6 mRNA expression. (d) The representative figures of WT-1 protein expression (magnification 400X; scale bar 100 m). ^*∗*^<0.01 vs. control, ^∫^^∫^<0.05 vs. DM1, and ^#^<0.01 vs. DM2.

Early treatment of CeA in DM might attenuate oxidative stress and podocyte injury with upregulation of SOD-1, SOD-3, and nephrin mRNA expression Figures 2 and 3. DM promotes activation of both enzymatic and nonenzymatic pathways that leads to ROS production. Activation of the AGE and its ligand and receptor, receptor advance glycated-end product (RAGE), in the endothelial surface leads to proinflammatory cytokines and free radicals productions [3, 46–48]. Control glucose loss inside the cell stimulates transport-chain electron dysfunction in the mitochondria that contributes to the biggest source of ROS [3, 5, 48, 49]. Antioxidant enzyme SOD has an essential role in catalyzing superoxide anion (*o*_2_^−^) into hydrogen peroxide and molecular oxygen, thus protecting cellular and histological damage from ROS [50, 51]. However, during the hyperglycemia, the SOD is unable to eliminate the excess ROS, which results in suppression of the antioxidant enzymes such as SOD1 [52,53], SOD3 [53], and catalase [52] but not SOD2. Reduction of the kidney SOD enzymes was demonstrated in KK/Ta-Akita mice after 5-week hyperglycemia mediated by TNF-*α* and interleukin-1*β* (IL-1*β*) [53]. Overexpression of SOD1 in the SOD1-Tg mice suppressed lipid peroxidation in the maternal hyperglycemia that leads to diminished susceptibility of diabetic embryopathy [53, 54]. Knockout of SOD3 resulted in reduced basal NO activity and increased superoxides in the endothelial and vascular that then led to impaired endothelial relaxation [55]. Oral treatment of CeA improves antioxidant enzymes by preventing lipid peroxidation [56], activates PPAR*γ* that then promotes insulin sensitivity, and represses TNF-*α* and NF*κ*B [37].

Hyperglycemic condition also induces podocyte injury. The detachment of the podocytes in the urinary sediment can be seen in patients with DM that precedes the proteinuria [56–58]. The diminished mRNA and protein level of nephrin in the DN-induced adriamycin alter the podocyte structure and integrity [33]. We showed STZ-induced DM markedly reduced the mRNA nephrin expression (Figure 2). The TRPC6 may play a key role in podocyte injury during DN. Knockout TRPC-6 in Akita mice attenuated glomerulosclerosis, tubular injury, and proteinuria while even promoting mesangial cell expansion [14, 59]. ROS induces glomerulosclerosis with the upregulation of the TRPC6 in podocytes [59, 60]. Podocyte culture transfected with scrambled siRNA targeted TRPC6 and Syndecan4 (Syn4), exposure to high d-glucose increased ROS, and TRPC6 via Syn4 [19]. Besides, the upregulation of the TRPC6 mRNA expression was observed in the monocytes of patients with type-2 DM that promotes atherosclerosis [61]. Based on our data, attenuation of podocyte injury may be associated with reducing hyperglycemia, upregulation of SOD-1/SOD-3 mRNA expression, and downregulation of the TRPC6 mRNA expression in the CeA treatment in early hyperglycemia condition (Figures 2 and 3). However, our study cannot correlate precisely the podocyte detachment with the reduction of podocyte marker expression in our study. The underlying mechanism of podocyte detachment may give a better understanding of the mechanism.

**Figure 4:**
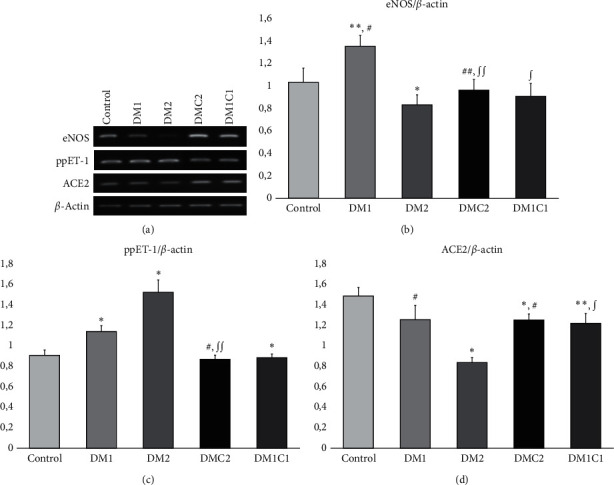
*Centella asiatica* preserved eNOS and down-regulated ACE2 and ppET-1 mRNA expression in DMC2 compared to DM2 groups. (a) The representative pictures of eNOS, ACE2, and ppET-1 based on RT-PCR quantification. (b -d) The bar charts of semiquantitative analysis of eNOS, ppET-1, and ACE2 mRNA expression. ^*∗*^<0.01 vs. control, ^*∗∗*^<0.05 vs. control, ^∫^<0.01 vs. DM1, ^∫^^∫^<0.05 vs. DM1, ^#^<0.01 vs. DM2, and ^##^<0.01 vs. DM2.

In this study, we observed that STZ-induced DM rat model promoted upregulation of ET-1 mRNA expression and downregulation of eNOS and ACE2 mRNA expressions (Figure 4). High production of ROS in DM plays an essential role in endothelial dysfunction through the inactivation of nitrite oxide (NO) and activation of renin-angiotensin-system (RAS). The eNOS has an essential role as an antiatherogenic that has a significant relevancy with the development of vascular injury in diabetes. The eNOS knockout mice demonstrated severe diabetic nephropathy concomitant with high blood pressure [62] and vascular hypertrophy [63]. Furthermore, deletion of the ET-1 in endothelial cells reduced oxidative stress after kidney ischemic/reperfusion injury (IRI) [30] which may correlate with the downregulation of SOD3 mRNA expression (Figure 2). ET-1 shows different effects through the Endothelin-A receptor (EDNRA) and Endothelin-B receptor (EDNRB) that exert various impacts. Several studies showed that the correlation between an increase of plasma ET-1 and elevation of GFR, mesangial cell expansion, and proteinuria [27]. Enhanced ET-1 level promotes vascular dysfunction via inhibition of the NO production that then leads to insufficient eNOS bioavailability and production [64]. The role of ET-1 promotes glomerulosclerosis is mediated by EDNRB; the administration of BQ-788, EDNRB antagonist, showed downregulation of the ET-1-induced calcium transient pathway that leads to podocyte detachment [65].

It has been demonstrated that RAS can play a role through the ACE-angiotensin II axis and ACE2-Ang1-7 axis. Angiotensin II raises renal ET-1 formation from podocyte cells and drives glomerulosclerosis and podocyte detachment [64, 65]. On the other hand, angiotensin II cleaved by angiotensin-converting enzyme2 (ACE2) produces Ang1-7 that exhibits a vasodilatation effect [29]. This enzyme significantly decreases in the DM condition as shown by the DM2 group. The pharmacological administration of ACE2 inhibitor elicited albuminuria and associated with the severity of the glomerular lesions [29]. The antioxidant effect from CeA is associated with the diminishment of ET-1 mRNA expression followed by the enhancement of eNOS and ACE2 mRNA expression.

**Figure 5:**
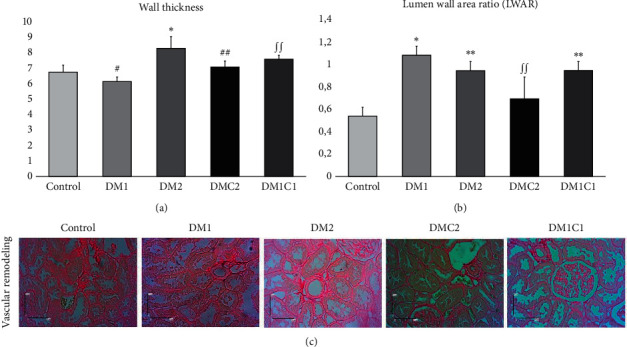
Ethanolic extract of *Centella asiatica* treatment in DMC2 group attenuated vascular remodeling with reducing wall thickness. (a, b) The quantification of wall area and wall/lumen area ratio which represented vascular remodeling measurement. (c) Representative pictures of intra-renal arteries for vascular remodeling assessment. ^*∗*^<0.01 vs. control, ^*∗∗*^<0.05 vs. control, ^∫^^∫^<0.05 vs. DM1, ^#^<0.01 vs. DM2, and ^##^<0.01 vs. DM2.

The early stage of DM showed hyperfiltration marked by vascular hypertrophy, and vascular injury [59]. An elevation of the GFR, one of the characteristics of early diabetes, correlates with an increase of vessel diameter and renal blood flow [66]. Our findings were consistent with the previous research that showed the dilatation of the lumen, basal membrane thickening, and increasing wall thickness (Figure 5). We demonstrated that the CeA treatment since the early hyperglycemia condition of the DM reduced the vascular lumen area and wall thickness area. However, the effect of CeA extract treatment during late hyperglycemia condition may not demonstrate kidney injury attenuation.

In this study, we want to focus on the protective effect of CeA in reducing the progression of DM in the early and late stage of diabetes mellitus. We explore several aspects, including oxidative stress, podocyte injury, glomerulosclerosis, and endothelial injury that become our main focus. Despite this, there are limitations in this study. One important limitation is that this study did not provide a positive control group which might not be compared with standardized therapeutics drugs, such as glibenclamide [67, 68], and metformin [69]. Even though our study reported that CeA treatment at the early stage of diabetes mellitus could improve random blood glucose level, glomerulosclerosis, antioxidant level, podocyte injury, and diabetic nephropathy, we cannot elucidate the effectiveness of CeA compared to the standardized therapeutic drugs. Besides, we did not provide the quality control of the extract. We are fully aware that the quality control process provides more information for the reader. Therefore, we cannot determine which active compound of CeA gives an essential role as a reno-protective agent in DM or the hazardous effect. In the future, these limitations need further investigation.

## 5. Conclusions

*Centella asiatica* (CeA) treatment at the early stage of DM ameliorates glomerulosclerosis and vascular injury via increasing antioxidant enzymes. However, treatment of CeA at the middle stage of DM less effectively ameliorates glomerular and vascular injury in diabetes mellitus .

## Figures and Tables

**Table 1:** Primer sequences.

Gene	Forward primer (5′ ⟶ 3′)	Reverse primer (5′ ⟶ 3′)	Annealing temperature
SOD1	GCGGTGAACCAGTTGTGGTG	AGCCACATTGCCCAGGTCTC	55
SOD2	ATGTTGTGTCGGGCGGCGTGCAGC	GCGCCTCGTGGTACTTCTCCTCGGTG	57
SOD3	AGGCAGCTCAGAGGCTCTTT	GAGGTTCCACACCTGACAAGC	63
Nephrin	ACTCAGGCTGACATCTGGGAT	AGAGCTGGAATGACAGTGATGG	55
TRPC6	AAGTGAACGAAGGGGAGCTG	ACAGTCTCTCCCCAAGCTTTC	60
ACE2	GCCCAAAAGATGAACGAGGC	GACGCTTGATGGTCGCATTC	60
eNOS	CCGGCGCTACGAAGAATG	AGTGCCACGGATGGAAATT	55
ppET-1	GTCGTCCCGTATGGACTAGG	ACTGGCATCTGTTCCCTTGG	57
*β*-Actin	GCAGATGTGGATCAGCAAGC	GGTGTAAAACGCAGCTCAGTAA	53

The cDNA was mixed with Taq Master Mix (Promega, GoTaq Green, M7122) and primers and then incubated in 94 °C denaturation for 10 s, annealing (according to the table) for 30 sec, and extension 72 °C for 1 min final extension phase ending with the conditions of 72 °C for 10 minutes for 35 cycles. The PCR products were separated using 2% agarose gel along with 100 bp DNA ladder (SMOBio, DM2400). The expression of the genes was quantified with a densitometry analysis using the ImageJ software, and the mRNA expression of *β*-actin was used as the housekeeping genes.

## Data Availability

Data are available on request through contacting the corresponding author and can be assessed through the supplementary files.

## Supplementary Materials

Supplementary Materials Supplementary Table 1: specific antibody information for immunohistochemistry. Supplementary Table 2: specific primer pair information for RT-PCR.

## References

[1] Alberti K. G. M. M., Zimmet P. Z. (1998). Definition, diagnosis and classification of diabetes mellitus and its complications. Part 1: diagnosis and classification of diabetes mellitus. Provisional report of a WHO Consultation. *Diabetic Medicine*.

[2] Cade W. T. (2008). Diabetes-Related microvascular and macrovascular diseases in the physical therapy setting. *Physical Therapy*.

[3] Brownlee M. (2005). The pathobiology of diabetic complications: a unifying mechanism. *Diabetes*.

[4] Fukai T., Ushio-fukai M. (2011). Superoxide dismutases: role in redox signaling, vascular function, and diseases. *Antioxidants & Redox Signaling*.

[5] Arfian N., Setyaningsih W. A. W., Romi M. M., Sari D. C. R. (2019). Heparanase upregulation from adipocyte associates with inflammation and endothelial injury in diabetic condition. *BMC Proceedings*.

[6] Yilmaz M. I., Saglam M., Caglar K. (2006). The determinants of endothelial dysfunction in CKD: oxidative stress and asymmetric dimethylarginine. *American Journal of Kidney Diseases*.

[7] Miranda-díaz A. G., Pazarín-villaseñor L., Yanowsky-escatell F. G., Andrade-sierra J., Changes E. K. (2016). Oxidative stress in diabetic nephropathy with early chronic kidney disease. *Journal of Diabetes Research*.

[8] Peng J.-R., Lu T.-T., Chang H.-T., Ge X., Huang B., Li W.-M. (2016). Elevated levels of plasma superoxide dismutases 1 and 2 in patients with coronary artery disease. *BioMed Research International*.

[9] Mondola P., Damiano S., Sasso A., Santillo M. (2016). The Cu, Zn superoxide dismutase: not only a dismutase enzyme. *Front. Physiol.*.

[10] Minakawa A., Fukuda A., Sato Y. (2019). Podocyte hypertrophic stress and detachment precedes hyperglycemia or albuminuria in a rat model of obesity and type2 diabetes-associated nephropathy. *Scientific Reports*.

[11] Liu B., He X., Li S., Xu B., Birnbaumer L., Liao Y. (2017). Deletion of diacylglycerol-responsive TRPC genes attenuates diabetic nephropathy by inhibiting activation of the TGF*β*1 signaling pathway. *American Journal of Translational Research*.

[12] Su J., Li S.-J., Chen Z.-H. (2010). Evaluation of podocyte lesion in patients with diabetic nephropathy: wilms’ tumor-1 protein used as a podocyte marker. *Diabetes Research and Clinical Practice*.

[13] Kandasamy Y., Smith R., Lumbers E. R., Rudd D. (2014). Nephrin - a biomarker of early glomerular injury. *Biomarker Research*.

[14] Staruschenko A. (2019). TRPC6 in diabetic kidney disease: good guy or bad guy?. *Kidney International*.

[15] Doublier S., Salvidio G., Lupia E. (2003). Nephrin expression is reduced in human diabetic nephropathy: evidence for a distinct role for glycated albumin and angiotensin II. *Diabetes*.

[16] Jim B., Ghanta M., Qipo A. (2012). Dysregulated nephrin in diabetic nephropathy of type 2 diabetes: a cross sectional study. *PLoS One*.

[17] Graham S., Ding M., Sours-Brothers S., Yorio T., Ma J. X., Ma R. (2007). Downregulation of TRPC6 protein expression by high glucose, a possible mechanism for the impaired Ca^2+^ signaling in glomerular mesangial cells in diabetes. *American Journal of Physiology: Renal Physiology*.

[18] Menè P., Pugliese G., Pricci F., Di Mario U., Cinotti G. A., Pugliese F. (1993). High glucose inhibits cytosolic calcium signaling in cultured rat mesangial cells. *Kidney International*.

[19] Thilo F., Lee M., Xia S., Zakrzewicz A., Tepel M. (2014). High glucose modifies transient receptor potential canonical type 6 channels via increased oxidative stress and syndecan-4 in human podocytes. *Biochemical and Biophysical Research Communications*.

[20] Spinetti G., Kraenkel N., Emanueli C., Madeddu P. (2010). Diabetes and vessel wall remodelling: from mechanistic insights to regenerative therapies. *Cardiovascular Research*.

[21] Kolluru G. K., Bir S. C., Kevil C. G. (2012). Endothelial dysfunction and diabetes: effects on angiogenesis, vascular remodeling, and wound healing. *International Journal of Vascular Medicine*.

[22] Kuwabara A., Satoh M., Tomita N., Sasaki T., Kashihara N (2010). Deterioration of glomerular endothelial surface layer induced by oxidative stress is implicated in altered permeability of macromolecules in Zucker fatty rats. *Diabetologia*.

[23] Fakhruddin S., Alanazi W., Jackson K. E. (2017). Diabetes-induced reactive oxygen species: mechanism of their generation and role in renal injury. *Journal of Diabetes Research*.

[24] Dellamea B. S., Leitão C. B., Friedman R., Canani L. H. (2014). Nitric oxide system and diabetic nephropathy. *Diabetology & Metabolic Syndrome*.

[25] Qian Y., Feldman E., Pennathur S., Kretzler M., Brosius F. C. (2014). Mechanisms of glomerulosclerosis in diabetic nephropathy. *Diabetes*.

[26] Creager M. A., Lüscher T. F., Cosentino F., Beckman J. A. (2003). Diabetes and vascular disease. *Circulation*.

[27] Kalani M. (2008). The importance of endothelin-1 for microvascular dysfunction in diabetes. *Vascular Health and Risk Management*.

[28] Renna N. F., de las Heras N., Miatello R. M. (2013). Pathophysiology of vascular remodeling in hypertension. *International Journal of Hypertension*.

[29] Ribeiro-Oliveira A., Nogueira A. I., Pereira R. M., Boas W. W. V., dosSantos R. A. S., eSilva A. C. S. (2008). The renin – angiotensin system and diabetes: an update. *Vascular Health Risk Management*.

[30] Arfian N., Emoto N., Vignon-Zellweger N., Nakayama K., Yagi K., Hirata K.-i. (2012). ET-1 deletion from endothelial cells protects the kidney during the extension phase of ischemia/reperfusion injury. *Biochemical and Biophysical Research Communications*.

[31] Shakir Jamil S., Nizami Q., Salam M. (2007). *Centella asiatica* (Linn.) urban óa review. *Indian Journal of Natural Products and Resources*.

[32] Tang B., Zhu B., Liang Y. (2011). Asiaticoside suppresses collagen expression and TGF-*β*/Smad signaling through inducing Smad7 and inhibiting TGF-*β*RI and TGF-*β*RII in keloid fibroblasts. *Archives of Dermatological Research*.

[33] Wang Z., Liu J., Sun W. (2013). Effects of asiaticoside on levels of podocyte cytoskeletal proteins and renal slit diaphragm proteins in adriamycin-induced rat nephropathy. *Life Sciences*.

[34] Sari D. C. R., Aswin S., Susilowati R. (2014). Ethanol extracts of *Centella asiatica* leaf improves memory performance in rats after chronic stress via reducing nitric oxide and increasing Brain- Derived Neurotrophic Factor (BDNF) concentration. *International Journal of Psychology*.

[35] Xu X., Wang Y., Wei Z. (2017). Madecassic acid, the contributor to the anti-colitis effect of madecassoside, enhances the shift of Th17 toward Treg cells via the PPAR *γ*/AMPK/ACC1 pathway. *Nature*.

[36] Hsu Y.-M., Hung Y.-c., Hu L., Lee Y.-j., Yin M.-c. (2015). Anti-diabetic effects of madecassic acid and rotundic acid. *Nutrients*.

[37] Oates J. C., Reilly C. M., Crosby M. B., Gilkeson G. S. (2002). Peroxisome proliferator-activated receptor? agonists: potential use for treating chronic inflammatory diseases. *Arthritis & Rheumatism*.

[38] Wang N., Verna L., Chen N.-G. (2002). Constitutive activation of peroxisome proliferator-activated receptor-*γ* suppresses pro-inflammatory adhesion molecules in human vascular endothelial cells. *Journal of Biological Chemistry*.

[39] Kumar S., Kumar V., Prakash O. (2012). Antidiabetic and hypolipidemic activities of Kigelia pinnata flowers extract in streptozotocin induced diabetic rats. *Asian Pacific Journal of Tropical Biomedicine*.

[40] Sari D. C. R., Arfian N., Tranggono U., Setyaningsih W. A. W., Romi M. M., Emoto N. (2019). Hippocampal brain-derived neurotrophic factor (BDNF), tyrosine kinase B (TrkB) and extracellular signal-regulated protein kinase 1/2 (ERK1/2) signaling in chronic electrical stress model in rats. *Iran. Journal of Basic Medical Sciences*.

[41] Sasikala S., Lakshminarasaiah S., Naidu M. D. (2015). Antidiabetic activity of *Centella asiatica* on streptozotocin induced diabetic male albino rats. *World Journal Pharmarcy Science*.

[42] Dai H., Liu Q., Liu B. (2017). Research progress on mechanism of podocyte depletion in diabetic nephropathy. *Journal Diabetes Research*.

[43] Schena F. P., Gesualdo L. (2005). Pathogenetic mechanisms of diabetic nephropathy. *Journal of the American Society of Nephrology*.

[44] Zhu Q., Zeng J., Li J. (2020). Effects of compound *Centella* on oxidative stress and Keap1-Nrf2-ARE pathway expression in diabetic kidney disease rats. *Evidence-Based Complementary and Alternative Medicine*.

[45] Zhao Y., Shu P., Zhang Y. (2014). Effect of *Centella asiatica* on oxidative stress and lipid metabolism in hyperlipidemic animal models. *Oxidative Medicine and Cellular Longevity*.

[46] Maritim A. C., Sanders R. A., Watkins J. B. (2003). Diabetes, oxidative stress, and antioxidants: a review. *Journal of Biochemical and Molecular Toxicology*.

[47] Yamagishi S., Fukami K., Matsui T. (2015). Crosstalk between advanced glycation end products (AGEs)-receptor RAGE axis and dipeptidyl peptidase-4-incretin system in diabetic vascular complications. *Cardiovascular Diabetology*.

[48] Brownlee M. (2001). Biochemistry and molecular cell biology of diabetic complications. *Nature*.

[49] Zhao J., Jin H., Gao J. (2018). Serum extracellular superoxide dismutase is associated with diabetic retinopathy stage in Chinese patients with type 2 diabetes mellitus. *Disease Markers*.

[50] Tiwari B. K., Pandey K. B., Abidi A. B., Rizvi S. I. (2013). Markers of oxidative stress during diabetes mellitus. *Journal Biomark*.

[51] Ullah A. (2016). Diabetes mellitus and oxidative stress–A concise review. *Saudi Pharmaceutical Journal*.

[52] Sadi G., Sahin G. (2019). Modulation of renal insulin signaling pathway and antioxidant enzymes with streptozotocin-induced diabetes : effects of resveratrol. *Mediacal MDPI*.

[53] Fujita H., Fujishima H., Chida S. (2009). Reduction of renal superoxide dismutase in progressive diabetic nephropathy. *Journal of the American Society of Nephrology*.

[54] Weng H., Li X., Reece E. A., Yang P. (2012). SOD1 suppresses maternal hyperglycemia-increased iNOS expression and consequent nitrosative stress in diabetic embryopathy. *American Journal of Obstetrics and Gynecology*.

[55] Jung O., Marklund S. L., Geiger H., Pedrazzini T., Busse R., Brandes R. P. (2003). Extracellular superoxide dismutase is a major determinant of nitric oxide bioavailability. *Circulation Research*.

[56] Ramachandran V., Saravanan R. (2013). Asiatic acid prevents lipid peroxidation and improves antioxidant status in rats with streptozotocin-induced diabetes. *Journal of Functional Foods*.

[57] Kang Z., Zeng J., Zhang T. (2019). Hyperglycemia induces NF‐*κ*B activation and MCP‐1 expression via downregulating GLP‐1R expression in rat mesangial cells: inhibition by metformin. *Cell Biology International*.

[58] Reiser J., Altintas M. M. (2016). Podocytes [version 1; referees: 2 approved]. *F1000 Research*.

[59] Pourghasem M., Shafi H., Babazadeh Z. (2015). Histological changes of kidney in diabetic nephropathy. *Caspian Journal of internal Medicine*.

[60] Hall G., Wang L., Spurney R. F. (2019). TRPC channels in proteinuric kidney diseases. *Cells*.

[61] Wuensch T., Thilo F., Krueger K., Scholze A., Ristow M., Tepel M. (2010). High glucose–induced oxidative stress increases transient receptor potential channel expression in human monocytes. *Diabetes*.

[62] Kosugi T., Heinig M., Nakayama T., Matsuo S., Nakagawa T. (2010). eNOS knockout mice with advanced diabetic nephropathy have less benefit from renin-angiotensin blockade than from aldosterone receptor antagonists. *The American Journal of Pathology*.

[63] Savard S., Lavoie P., Villeneuve C., Agharazii M., Lebel M., Lariviere R. (2012). eNOS gene delivery prevents hypertension and reduces renal failure and injury in rats with reduced renal mass. *Nephrology Dialysis Transplantation*.

[64] Ozdemir B., Yazici A. (2020). Could the decrease in the endothelial nitric oxide (NO) production and NO bioavailability be the crucial cause of COVID-19 related deaths?. *Med. Hypotheses*.

[65] Lenoir O., Milon M., Virsolvy A. (2014). Direct action of Endothelin-1 on podocytes promotes diabetic glomerulosclerosis. *Journal of the American Society of Nephrology*.

[66] Awad Allah R. S., Dkhil M. A., Danfour M. A. (2007). Structural alterations of the glomerular wall and vessels in early stages of diabetes mellitus (light and transmission electron microscopic study). *Libyan Journal of Medicine*.

[67] Candasamy M., Murthy T. E. K., Gubiyappa K., Chellappan D., Gupta G. (2014). Alteration of glucose lowering effect of glibenclamide on single and multiple treatments with fenofibrate in experimental rats and rabbit models. *Journal of Basic and Clinical Pharmacy*.

[68] Bunyapraphatsara N., Yongchaiyudha S., Rungpitarangsi V., Chokechaijaroenporn O. (1996). Antidiabetic activity of Aloe vera L. juice II. Clinical trial in diabetes mellitus patients in combination with glibenclamide. *Phytomedicine*.

[69] Yoon S. H., Han E. J., Sung J. H., Chung S. H. (2007). Anti-diabetic effects of compound K versus metformin versus compound K-metformin combination therapy in diabetic db/db mice. *Biological and Pharmaceutical Bulletin*.

