# Supplementary material for: Ethanolic Extract of Centella asiatica Treatment in the Early Stage of Hyperglycemia Condition Inhibits Glomerular Injury and Vascular Remodeling in Diabetic Rat Model
Source: Evid Based Complement Alternat Med. 2021 Jul 6;2021:6671130. doi: 10.1155/2021/6671130 (PMC8277496; doi:10.1155/2021/6671130)
Supplement: Supplementary Materials — Supplementary Table 1: specific antibody information for immunohistochemistry. Supplementary Table 2: specific primer pair information for RT-PCR. [file 6671130.f1.docx]

Supplementary table 1. Specific antibody information for immunohistochemistry.

| Antibody | Subcellular location | Antibody types | Supplier names |
| --- | --- | --- | --- |
| anti-SOD1 antibody | Cytoplasm | Purified, Rabbit polyclonal antibody | Bioss (Massachusetts, USA) |
| anti-WT-1 antibody | Nuclear | Purified, Rabbit polyclonal antibody | Santa Cruz (Texaz, USA) |

SOD1=Superoxide dismutase1; WT-1=Wilms tumor-1

Supplementary table 2. Specific primer pairs information for RT-PCR.

| Gene | Forward primer (5′ 🡪 3′) | Reverse primer (5′ 🡪 3′) | Product size (bp) | Accession number |
| --- | --- | --- | --- | --- |
| SOD1 | GCGGTGAACCAGTTGTGGTG | AGCCACATTGCCCAGGTCTC | 191 | [NM_017050.1](https://www.ncbi.nlm.nih.gov/entrez/viewer.fcgi?db=nucleotide&id=8394327) |
| SOD2 | ATGTTGTGTCGGGCGGCGTGCAGC | GCGCCTCGTGGTACTTCTCCTCGGTG | 217 | [NM_017051.2](https://www.ncbi.nlm.nih.gov/entrez/viewer.fcgi?db=nucleotide&id=47575854) |
| SOD3 | AGGCAGCTCAGAGGCTCTTT | GAGGTTCCACACCTGACAAGC | 100 | [NM_012880.1](https://www.ncbi.nlm.nih.gov/entrez/viewer.fcgi?db=nucleotide&id=6981567) |
| Nephrin | ACTCAGGCTGACATCTGGGAT | AGAGCTGGAATGACAGTGATGG | 299 | [NM_022628.1](https://www.ncbi.nlm.nih.gov/entrez/viewer.fcgi?db=nucleotide&id=12018317) |
| TRPC6 | AAGTGAACGAAGGGGAGCTG | ACAGTCTCTCCCCAAGCTTTC | 129 | [NM_053559.1](https://www.ncbi.nlm.nih.gov/entrez/viewer.fcgi?db=nucleotide&id=16758329) |
| ACE2 | GCCCAAAAGATGAACGAGGC | GACGCTTGATGGTCGCATTC | 112 | [NM_001012006.1](https://www.ncbi.nlm.nih.gov/entrez/viewer.fcgi?db=nucleotide&id=58865587) |
| eNOS | CCGGCGCTACGAAGAATG | AGTGCCACGGATGGAAATT | 78 | [NM_021838.2](https://www.ncbi.nlm.nih.gov/entrez/viewer.fcgi?db=nucleotide&id=46409655) |
| ppET-1 | GTCGTCCCGTATGGACTAGG | ACTGGCATCTGTTCCCTTGG | 100 | [NM_012548.2](https://www.ncbi.nlm.nih.gov/entrez/viewer.fcgi?db=nucleotide&id=292658816) |
| $\boldsymbol{\beta}$-actin | GCAGATGTGGATCAGCAAGC | GGTGTAAAACGCAGCTCAGTAA | 100 | [NM_031144.3](https://www.ncbi.nlm.nih.gov/entrez/viewer.fcgi?db=nucleotide&id=402744873) |

SOD1=Superoxide dismutase1; SOD2=Superoxide dismutase2; SOD3=Superoxide dismutase3;TRPC6= canonical transient receptor potential channel type 6 ;ACE2=angiotensin-converting enzymes2; eNOS=endothelial nitric oxide synthase; ppET-1=prepreEndothelin-1; $\beta$-actin=beta-actin
